# Joint association of estimated skeletal muscle mass index and prognostic nutritional index with all-cause mortality in individuals with non-metastatic nasopharyngeal cancer

**DOI:** 10.3389/fnut.2026.1768802

**Published:** 2026-05-14

**Authors:** Fansu Huang, Cai Gong, Huan Luo, Qin Wang, Yanming Hu, Ruzhe Zhang, Yanni Hu, Tao Hou, Yangchun Xie

**Affiliations:** 1 Department of Clinical Nutrition, The Second Xiangya Hospital, Central South University, Changsha, Hunan, China; 2 Department of Radiology, Zhangjiajie People’s Hospital, Zhangjiajie, Hunan, China; 3 Department of Oncology, The Second Xiangya Hospital, Central South University, Changsha, Hunan, China

**Keywords:** all-cause mortality, cohort, estimated skeletal muscle mass index, nasopharyngeal cancer, prognostic nutritional index

## Abstract

**Background:**

The prognostic interplay between skeletal muscle mass and nutritional/immune status in non-metastatic nasopharyngeal carcinoma (NPC) is unclear. This study investigates the independent and joint association of estimated skeletal muscle mass index (eSMI) and Prognostic Nutritional Index (PNI) with all-cause mortality.

**Methods:**

We analyzed 942 non-metastatic NPC patients. Low muscle mass was defined by sex-specific 20th percentiles of eSMI. PNI was categorized using a clinical cutoff of 45. Patients were stratified into four groups: normal eSMI & high PNI (reference), normal eSMI & Low PNI, low eSMI & high PNI, and low eSMI & low PNI. Multivariate Cox models and a win ratio analysis were used to evaluate joint prognostic value.

**Results:**

During a median follow-up of 54 months, 204 deaths (21.7%) occurred. In multivariate Cox analysis, low PNI was independently associated with higher risk (HR = 1.68, *p* = 0.006), while low eSMI alone was not (HR = 1.05, 95% CI: 0.66–1.66). However, the low eSMI & low PNI group exhibited the highest mortality risk (HR = 2.39, 95% CI: 1.32–4.30, *p* = 0.004). The win ratio analysis confirmed a significant clinical benefit for the normal SMI & high PNI over the low SMI & low PNI group, with a win ratio of 1.547 (95% CI: 1.54–1.56, *p* < 0.001).

**Conclusion:**

Joint assessment of eSMI and PNI provides superior risk stratification. Pre-treatment assessment of these simple markers may facilitate targeted nutritional and supportive interventions.

## Introduction

Nasopharyngeal carcinoma (NPC) is a malignant epithelial cancer of the nasopharyngeal lining, notable for its high metastatic potential among head and neck cancers. It exhibits a notably high prevalence in southern China and Southeast Asia, with age-standardized rates of 4–25 cases per 100,000 individuals (1, 2). Despite significant advancements in prevention, early screening, and treatments such as radiotherapy and chemoradiotherapy, NPC continues to pose a substantial burden on public health systems (3, 4). The standard treatments often cause nutritional insufficiency, skeletal muscle loss, and impaired immunonutrition, which collectively undermine treatment efficacy and long-term survival (5–8). Malnutrition is linked to poorer treatment responses, greater toxicity, and reduced overall survival (9–15). Accordingly, evaluating metabolic reserve (skeletal muscle mass) and immunonutritional status has become essential for precise prognostic stratification in NPC.

Skeletal muscle, the body’s largest organ, serves as a pivotal reservoir for metabolic homeostasis and immune function. Accumulating clinical evidence has delineated close interconnections among reduced muscle mass, malnutrition, and cancer-related cachexia (10, 11, 16–18). Specifically, low skeletal muscle mass, or sarcopenia, has been consistently linked to malnutrition, cachexia development, and compromised survival outcomes in patients with various cancers, including nasopharyngeal carcinoma. Notably, cancer cachexia not only accelerates progressive muscle wasting but also is largely irreversible with conventional nutritional interventions (19), creating a vicious cycle that further exacerbates poor clinical outcomes. This highlights the critical need for early identification of skeletal muscle loss in the clinical management of NPC patients. However, the gold-standard muscle mass assessment techniques such as MR, computed tomography (CT) and dual-energy X-ray absorptiometry (DXA) are largely limited in routine clinical practice due to high cost or limited accessibility. Fortunately, the anthropometric formula developed by Wen et al. (20) for Chinese adults provides a validated, non-invasive surrogate that correlates strongly with DXA-measured skeletal muscle mass.

On the other hand, the host’s immunonutritional status, captured by the Prognostic Nutritional Index (PNI), reflects systemic inflammation and nutritional reserve. It incorporates serum albumin levels and peripheral blood lymphocyte counts, has been repeatedly validated for its prognostic value in various cancers (19, 21, 22). A large meta-analysis confirmed that low baseline PNI was an independent predictor of poor overall and progression-free survival in these patients (23). Other clinical studies have also found that low pre-treatment PNI is closely linked to heightened treatment-related toxicities, which can severely impair patient tolerance and lead to higher rates of treatment interruption (24, 25). Despite the established role of muscle mass and PNI in cancer prognosis, their potential synergistic and interactive effects in non-metastatic NPC have not been fully elucidated to date. To address this gap, this study aims to investigate the independent and joint association of muscle mass and Prognostic Nutritional Index (PNI) with all-cause mortality in NPC patients.

## Materials and methods

### Study design and population

**Figure 1:**
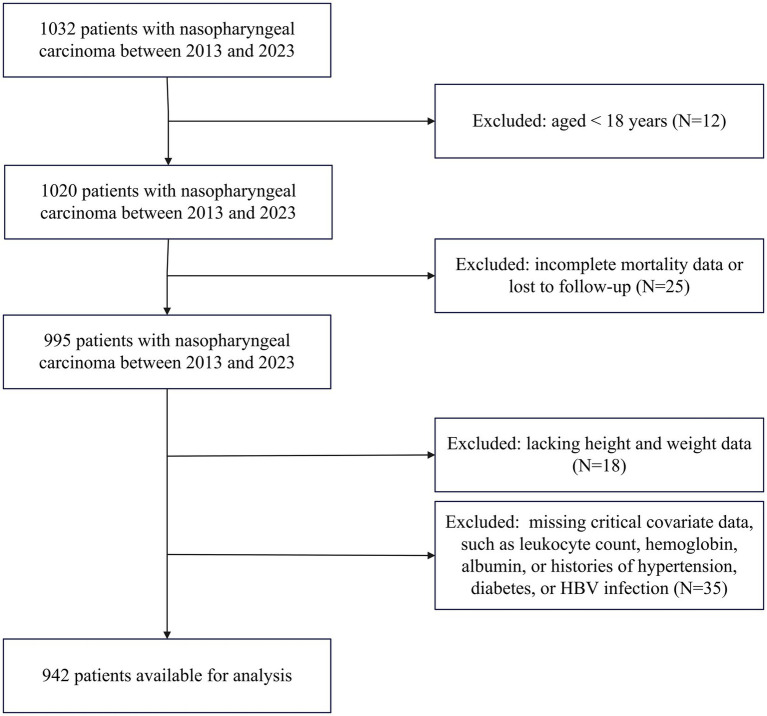
The flow chart of participant selection.

This retrospective cohort study was conducted in the Department of Oncology at a tertiary care hospital in China. Patients diagnosed with non-metastatic nasopharyngeal carcinoma between 2013 and 2023 were screened. Exclusion criteria included (1) patients under 18 years of age (*N* = 12), (2) those with incomplete mortality data or lost to follow-up (*N* = 25), (3) those lacking complete height and weight data (*N* = 18), and (4) patients missing critical covariate data, such as leukocyte count, hemoglobin, albumin, or histories of hypertension, diabetes, or HBV infection (*N* = 35). Following exclusions, 942 participants were included in the analysis (Figure 1). The protocol of this study was approved by the ethics committee of the Second Xiangya Hospital of Central South University (LYEC2024-0321). The study followed STROBE guidelines.

### Demographic and laboratory data

Data collected included age, sex, height, weight, smoking and drinking status, leukocyte count, neutrophil count, hemoglobin, and albumin levels. Clinical information including clinical staging, use of concurrent chemoradiotherapy (CCRT), performance status (PS) score was also collected. Family history of cancer and histories of hypertension, diabetes, and Hepatitis B virus (HBV) infection were also recorded. All data were collected within one-week prior anti-tumor treatment. Body mass index (BMI) was calculated as weight (kg) divided by height squared (m^2^).

### Skeletal muscle and PNI assessment

Appendicular skeletal muscle mass (ASM) was estimated using the Wen et al. formula (20): *ASM = body weight (kg) × 0.193 + height (m) × 0.107–4.157 × sex (where 1 = male, 2 = female) − 0.037 × age (years) − 2.631*. The estimated skeletal muscle mass index (eSMI) was then calculated by dividing ASM by the square of height in meters [eSMI = ASM (kg)/height^2^ (m^2^)]. To avoid gender bias, low SMI was defined by the sex-specific 20th percentile (P20) of the cohort (20, 26): <7.26 kg/m^2^ in males and <5.71 kg/m^2^ in females. PNI was calculated (19) using the formula: PNI = ALB (g/L) + 5 × total lymphocyte count (10^9^/L). A cutoff of 45 based on previous studies was used to define low PNI (23). Participants were then categorized into four joint groups: normal SMI & high PNI, normal SMI & low PNI, low SMI & high PNI, and low SMI & low PNI.

### Outcomes

The primary outcome was all-cause mortality. Survival status was determined through telephone interviews conducted every 12 months following the baseline evaluation. For participants who died, the time from baseline to the date of death was recorded. For those still alive at the end of the study, the time from baseline to the last contact was used. Overall survival (OS) was defined as the time from the first visit to either death or last follow-up.

### Statistical analyses

Categorical variables were presented as numbers and percentages, while continuous variables were expressed as mean ± standard deviation (SD) or median ± interquartile range based on distribution. Pearson’s chi-squared test was applied to categorical variables, one-way ANOVA for normally distributed continuous variables, and the Mann–Whitney U test for non-normally distributed continuous variables.

To explore the independent and joint associations of SMI and PNI with all-cause mortality, Kaplan–Meier survival curves were conducted with differences assessed via the log-rank test. In addition, univariate and multivariate Cox proportional hazards models were performed. We used two sequentially adjusted multivariate models: Model 1 for age and sex; Model 2 for additional adjustment of smoking status, drinking status, chronic disease comorbidities (histories of hypertension, diabetes, or HBV infection), clinical staging, pathological T and N stages, CCRT, PS score, neutrophil count, eSMI, PNI and BMI. Variance Inflation Factor (VIF) was used to detect multicollinearity (VIF > 10 defined as severe multicollinearity). For continuous eSMI analysis, BMI was excluded from Model 2 due to severe multicollinearity (VIF = 60); for grouped eSMI-PNI analysis, all covariates (including BMI) were retained as no multicollinearity was observed. Results were presented as the form of hazard ratios (HRs) and 95% confidence intervals (CI).

To address the limitations of single-outcome Cox models, we performed an Inverse Probability of Treatment Weighting (IPTW)-adjusted win ratio analysis (27, 28), with hierarchical endpoint prioritization: all-cause mortality > disease progression. Weighted pairwise comparisons of all patient pairs between the low-risk (normal eSMI & high PNI) and high-risk (low eSMI & low PNI) groups were conducted. The win ratio (WR), win odds, and net benefit were calculated to provide a multifaceted evaluation of clinical outcome differences. Win ratio (WR) > 1 indicates that the low-risk group was associated with a superior clinical outcome (lower all-cause mortality first, then less disease progression) relative to the high-risk group.

Subgroup analyses were further performed to verify these associations across key clinical subgroups. Participants were stratified by age (<45 vs. ≥45 years), BMI (≥24 vs. <24 kg/m^2^), comorbidities status (yes vs. no), the use of CCRT (yes vs. no) and PS score (0 vs. 1–2). Statistical significance was determined at a *p* value < 0.05. All statistical analyses were performed via R version 4.4.1.

## Results

### Characteristics of subjects

A total of 942 patients were enrolled, with a mean age of 49 ± 10 years, mean eSMI of 7.38 ± 0.94 kg/m^2^, and mean PNI of 50.3 ± 4.7. Table 1 displays the baseline characteristics stratified by four joint eSMI-PNI groups. The groups were well balanced in terms of gender, smoking/drinking status, family history of cancers, chronic disease comorbidities, tumor T/N/clinical stage, and CCRT administration (all *p* > 0.05). Significant intergroup differences were observed in age, PS score, BMI, hemoglobin, albumin, eSMI, PNI, with the normal eSMI & low PNI group and low eSMI & low PNI group showing the most unfavorable profiles (higher proportion of poor PS score, as well as lower levels of hemoglobin and albumin). Stratification by eSMI alone (Supplementary Table S1) or PNI alone (Supplementary Table S2) further confirmed good baseline balance in major clinical and therapeutic covariates among subgroups.

**Table 1:** Characteristics of the population by joint groups of eSMI and PNI.

Characteristic of baseline	Overall *N* = 942	Normal eSMI & high PNI *N* = 677	Normal eSMI & low PNI *N* = 75	Low eSMI & high PNI *N* = 153	Low eSMI & low PNI *N* = 37	*p*-value^1^
Male	276 (29%)	198 (29%)	22 (29%)	46 (30%)	10 (27%)	0.987
Age, years	49 ± 10	48 ± 10	48 ± 10	53 ± 10	52 ± 10	**<0.001**
Age group						**0.006**
<45 years	306 (32%)	240 (35%)	24 (32%)	32 (21%)	10 (27%)	
≥45 years	636 (68%)	437 (65%)	51 (68%)	121 (79%)	27 (73%)	
Current smoking	229 (24%)	163 (24%)	20 (27%)	36 (24%)	10 (27%)	0.931
Current drinking	141 (15%)	98 (14%)	10 (13%)	25 (16%)	8 (22%)	0.615
Family history of cancers	30 (3.2%)	23 (3.4%)	3 (4.0%)	4 (2.6%)	0 (0%)	0.785
Comorbidity of chronic diseases^2^	348 (37%)	258 (38%)	27 (36%)	53 (35%)	10 (27%)	0.506
T stage						0.348
1–2	492 (52%)	360 (53%)	32 (43%)	82 (54%)	18 (49%)	
3–4	450 (48%)	317 (47%)	43 (57%)	71 (46%)	19 (51%)	
N stage						0.739
0–1	222 (24%)	165 (24%)	18 (24%)	31 (20%)	8 (22%)	
2–3	720 (76%)	512 (76%)	57 (76%)	122 (80%)	29 (78%)	
Clinical stage						0.268
I–II	131 (14%)	99 (15%)	7 (9.3%)	20 (13%)	5 (14%)	
III	583 (62%)	406 (60%)	47 (63%)	107 (70%)	23 (62%)	
IV	228 (24%)	172 (25%)	21 (28%)	26 (17%)	9 (24%)	
OS, months	54 (37, 75)	54 (37, 77)	52 (35, 76)	58 (38, 72)	48 (33, 67)	0.271
CCRT	289 (31%)	218 (32%)	19 (25%)	41 (27%)	11 (30%)	0.416
PS score						**<0.001**
0	548 (58%)	428 (63%)	33 (44%)	71 (46%)	16 (43%)	
1–2	394 (42%)	249 (37%)	42 (56%)	82 (54%)	21 (57%)	
BMI, kg/m^2^	23.4 ± 3.2	24.5 ± 2.6	23.7 ± 2.6	19.3 ± 1.4	19.2 ± 1.5	**<0.001**
BMI group						**<0.001**
<24 kg/m^2^	547 (58%)	313 (46%)	44 (59%)	153 (100%)	37 (100%)	
≥24 kg/m^2^	395 (42%)	364 (54%)	31 (41%)	0 (0%)	0 (0%)	
Hemoglobin, g/L	142 ± 16	144 ± 15	129 ± 13	138 ± 15	126 ± 13	**<0.001**
eSMI, kg/m^2^	7.38 ± 0.94	7.61 ± 0.85	7.45 ± 0.81	6.50 ± 0.76	6.54 ± 0.77	**<0.001**
PNI	50.3 ± 4.7	51.4 ± 3.8	42.6 ± 2.6	50.9 ± 4.0	42.5 ± 2.4	**<0.001**
Albumin, g/L	41.8 ± 3.5	42.6 ± 2.9	36.4 ± 2.4	42.1 ± 3.2	36.0 ± 2.2	**<0.001**
Albumin group						**<0.001**
<40 g/L	271 (29%)	124 (18%)	73 (97%)	38 (25%)	36 (97%)	
≥40 g/L	671 (71%)	553 (82%)	2 (2.7%)	115 (75%)	1 (2.7%)	
Leukocyte, 10^9^/L	6.60 ± 1.89	6.72 ± 1.87	5.88 ± 1.81	6.50 ± 1.78	6.43 ± 2.60	**<0.001**
Neutrophil, 10^9^/L	4.37 ± 1.66	4.42 ± 1.60	4.13 ± 1.71	4.22 ± 1.61	4.59 ± 2.50	0.100
Lymphocyte, 10^9^/L	1.70 ± 0.58	1.76 ± 0.57	1.23 ± 0.35	1.76 ± 0.61	1.30 ± 0.36	**<0.001**

The continuous data were presented as the mean ± SD or median (IQR) as appropriate. The categorical data were presented as absolute numbers and percentages (%) of the total.

^1^Pearson’s Chi-squared test, Kruskal-Wallis rank sum test or Fisher’s exact test were used as appropriate.

^2^Comorbidity of chronic diseases was determined by the histories of hypertension, diabetes, or HBV infection.

BMI, body mass index; CCRT, concurrent chemoradiotherapy; HBV, hepatitis B virus; OS, overall survival; PNI, prognostic nutritional index; PS, performance status; SD, standard deviation; eSMI, estimated skeletal muscle index. Bold values indicate statistically significant differences (*p* < 0.05).

### Associations among SMI, PNI, and mortality

**Figure 2:**
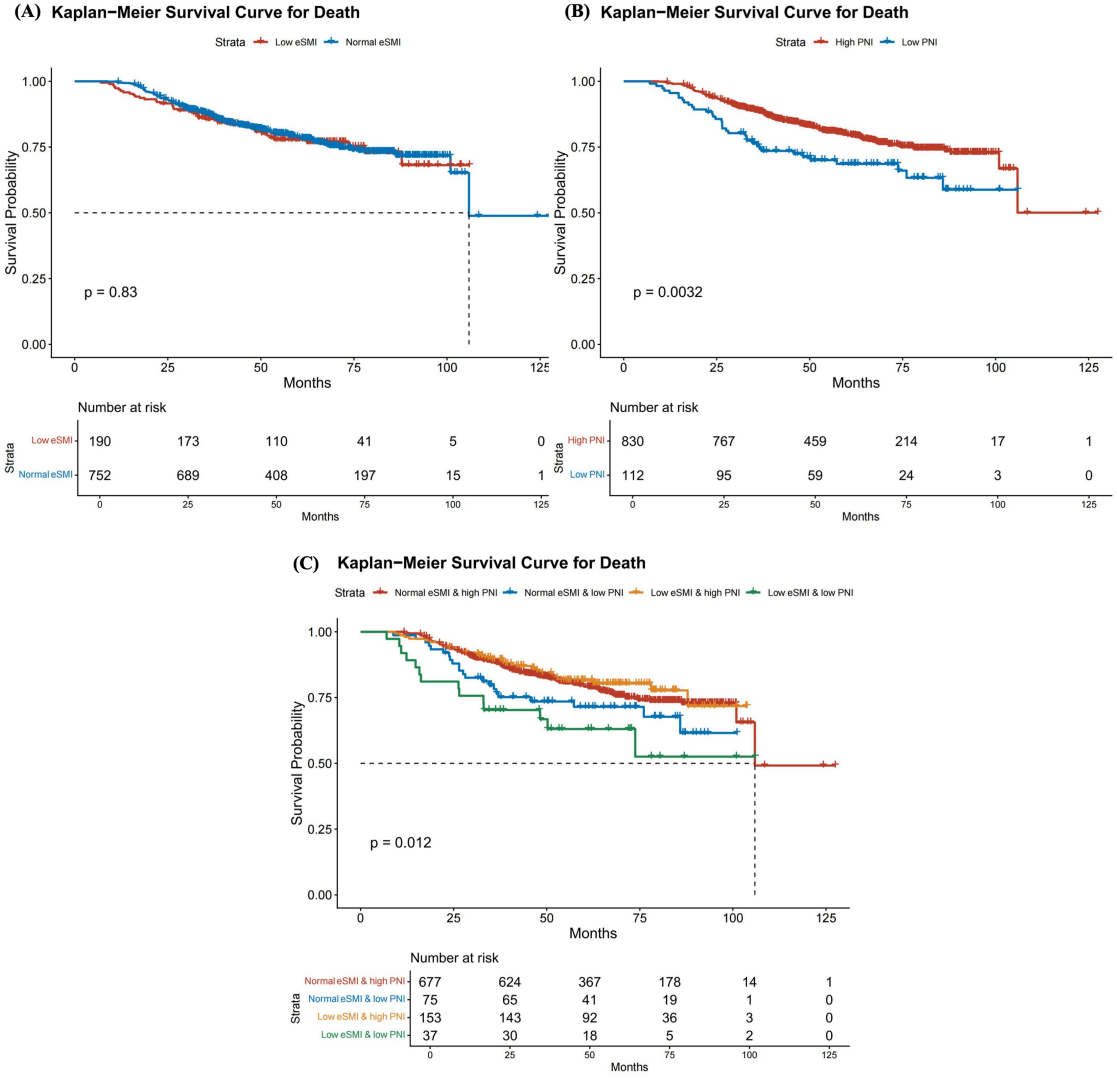
Kaplan–Meier curves of the survival rate of participants with different eSMI and PNI levels. **(A)** Comparison of survival rates between participants with normal SMI and low SMI. **(B)** Comparison of survival rates between participants with PNI ≥ 45 and PNI < 45. **(C)** Comparison of survival rates among four groups: high eSMI & high ePNI, high eSMI & low PNI, low eSMI & high PNI, and low eSMI & low PNI. eSMI, estimated skeletal muscle index; PNI, prognostic nutritional index.

During a median follow-up of 54 months, a total of 204 deaths (21.7%) were recorded. In the Cox analysis (Table 2), eSMI alone showed no significant association with all-cause mortality in the fully adjusted Model 2 (HR = 1.05, 95%CI: 0.66–1.66, *p* = 0.851), which was consistent with the Kaplan–Meier survival analysis (Figure 2A, *p* = 0.830). In contrast, low PNI (<45) was identified as a robust independent prognostic risk factor for all-cause mortality. After full adjustment, low PNI still conferred a 68% increased risk of death compared with high PNI (≥45) (HR = 1.68, 95%CI: 1.16–2.44, *p* = 0.006), and Kaplan–Meier curves demonstrated significantly poorer survival outcomes in the low PNI group (Figure 2B, *p* = 0.003).

**Table 2:** Cox regression for all-cause mortality based on different eSMI and PNI levels.

Character	Crude model		Model 1		Model 2	
	HR (95%CI)	*p*-value	HR (95%CI)	*p*-value	HR (95%CI)	*p*-value
eSMI, kg/m^2^	0.88 (0.76, 1.02)	0.090	1.00 (0.81, 1.25)	0.973	1.00 (0.80, 1.25)^#^	0.983
eSMI group						
Normal	Ref		Ref		Ref	
Low	1.04 (0.74, 1.46)	0.833	1.04 (0.74, 1.46)	0.830	1.05 (0.66, 1.66)	0.851
PNI	0.96 (0.93, 0.99)	**0.010**	0.96 (0.93, 0.99)	**0.009**	0.96 (0.93, 0.99)	**0.007**
PNI group						
≥45	Ref		Ref		Ref	
<45	1.71 (1.19, 2.45)	**0.004**	1.72 (1.20, 2.46)	**0.003**	1.68 (1.16, 2.44)	**0.006**
Joint group						
Normal eSMI & high PNI	Ref		Ref		Ref	
Normal eSMI & low PNI	1.47 (0.94, 2.31)	0.092	1.47 (0.94, 2.31)	0.090	1.51 (0.95, 2.40)	0.081
Low eSMI & high PNI	0.87 (0.58, 1.31)	0.508	0.87 (0.58, 1.31)	0.506	0.96 (0.61, 1.52)	0.878
Low eSMI & low PNI	2.11 (1.22, 3.67)	**0.008**	2.14 (1.23, 3.74)	**0.007**	2.39 (1.32, 4.30)	**0.004**
*p* for trend		0.218		0.209		0.073

Model 1 was adjusted for age and sex.

Model 2 was further adjusted for smoking status, drinking status, comorbidities of chronic diseases, clinical staging, pathological T and N stage, CCRT, PS score, neutrophil, eSMI, PNI, and BMI.

^#^ BMI was excluded from Model 2 due to severe multicollinearity with continuous eSMI (VIF = 60).

BMI, body mass index; CCRT, concurrent chemoradiotherapy; PNI, prognostic nutritional index; PS, performance status; eSMI, estimated skeletal muscle index. Bold values indicate statistically significant differences (*p* < 0.05).

### Joint associations of SMI and PNI with mortality

**Figure 3:**
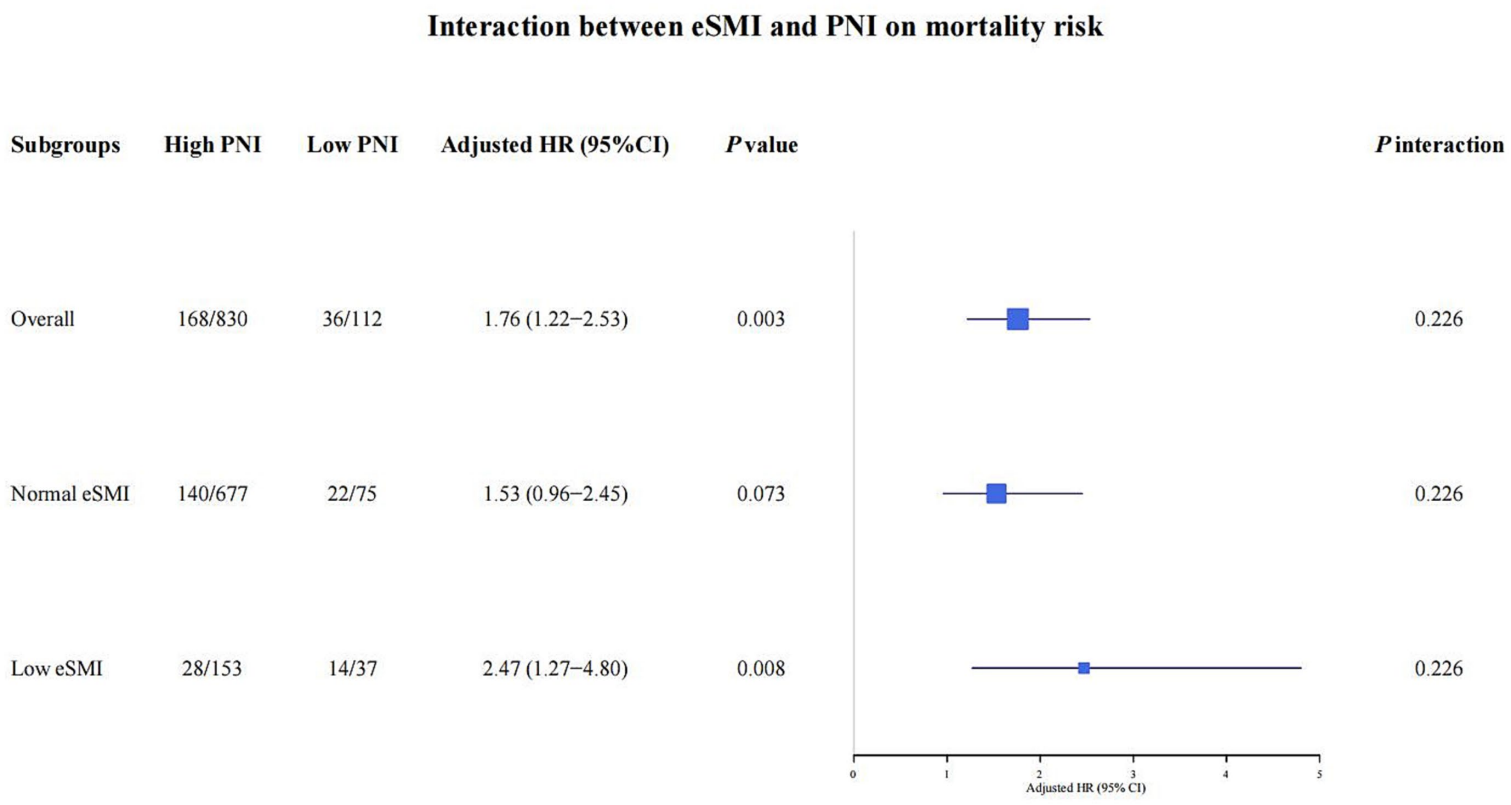
Interaction between eSMI and PNI on mortality risk. eSMI, estimated skeletal muscle index; PNI, prognostic nutritional index.

In the analysis of the joint eSMI-PNI groups, the low eSMI & low PNI group exhibited a 2.39-fold increase in mortality risk in the fully adjusted Model 2 (95% CI: 1.32–4.30, *p* = 0.004, Table 2) compared with the normal eSMI & high PNI group. Kaplan–Meier survival analysis for the four joint groups confirmed significant differences in overall survival (Figure 2C, *p* = 0.012). Although the interaction between eSMI and PNI on mortality was not statistically significant (*p* for interaction = 0.226, Figure 3), stratified analysis further demonstrated that low PNI was significantly associated with higher risk of all-cause mortality compared with high PNI in the low eSMI subgroup (adjusted HR = 2.47, 95%CI: 1.27–4.80, *p* = 0.008). The win ratio was 1.547 (95% CI: 1.536–1.557, *p* < 0.0001, Supplementary Table S3), indicating that patients in the normal eSMI & high PNI group were 54.7% more likely to have a better prioritized clinical outcome (avoiding death and then avoiding progression). These results highlight the synergistic adverse effect of combined low muscle mass and poor nutritional status.

### Subgroup analysis

Subgroup analysis (Supplementary Table S4) confirmed that the synergistic adverse effect of low eSMI combined with low PNI on mortality was significant in patients aged ≥45 years (HR = 2.69, 95%CI:1.33–5.41, *p* = 0.006), with BMI < 24 kg/m^2^ (HR = 2.31, 95%CI:1.27–4.18, *p* = 0.006) and without CCRT (HR = 3.12, 95%CI:1.58–6.17, *p* < 0.001), as well as those with chronic comorbidities (HR = 5.29, 95%CI:1.67–16.81, *p* = 0.005). In contrast, among patients who received CCRT, the prognostic impact of the joint markers did not reach statistical significance (HR = 1.59, 95% CI: 0.45–5.69, *p* = 0.475).

## Discussion

This study investigated the independent and joint associations of muscle mass and PNI with mortality risk in individuals with non-metastatic nasopharyngeal carcinoma. Our findings indicate that the concurrent presence of low eSMI and low PNI identifies a subgroup of patients at exceptionally high risk, exhibiting a more than two-fold increase in all-cause mortality. This is further validated by our win ratio analysis, which prioritized hierarchical clinical outcomes (death followed by progression). These results highlight that muscle and nutritional status complement tumor stage in stratifying mortality risk for NPC, supporting eSMI and PNI as a simple tool to identify high-risk patients in clinical settings.

The independent associations of eSMI and PNI with all-cause mortality exhibited distinct patterns in our non-metastatic NPC cohort. Estimated SMI showed no significant independent link to mortality in the fully adjusted model. In previous evidence, the prognostic value of muscle mass is highly heterogeneous. On one hand, low muscle mass has been widely validated as an independent adverse prognostic factor across cancers. Liu et al. (29) reported sarcopenia predicted poorer overall and progression-free survival in NPC (HR = 2.00 and 1.67, both *p* < 0.001), and a meta-analysis (11) confirmed pretreatment sarcopenia predicted inferior survival (HR = 3.09, *p* < 0.001). Contrarily, Ucgul et al. (30) found baseline sarcopenia correlated with progression-free but not overall survival in multivariable analysis. Another meta-analysis (31) further showed low muscle mass was not independently associated with overall survival in incurable cancers, except when combined with obesity. This inconsistency may reflect heterogeneous definitions, measurement standards, and cohort characteristics. In our non-metastatic cohort, relatively low tumor burden and preserved immunonutrition likely compensated for mild muscle loss, while assessment limited to muscle quantity (not quality or function) may have underestimated its prognostic effect. Future multi-center studies are needed to clarify the stage-specific and multi-dimensional prognostic role of muscle mass in NPC.

By contrast, PNI serves as the primary independent prognostic driver for survival in non-metastatic NPC, aligning with most NPC studies (24, 32), as it integrates nutritional reserve and immune function, both critical in EBV-driven, highly immunogenic NPC. Our results showed that low PNI (<45) was a robust independent risk factor, increasing mortality hazard by 68%. Low PNI remained strongly prognostic in the low eSMI subgroup (HR = 2.47, 95%CI: 1.27–4.80, *p* = 0.008), confirming its prognostic independence from muscle mass. Consistently, a meta-analysis (19) illustrated that PNI serves as a robust predictor of short- and long-term survival in NPC, with a pooled HR of 1.88 (95% CI: 1.10–3.20; *p* = 0.02) for low PNI on multivariate analysis. The prognostic utility of PNI extends beyond NPC. Zhang et al. (21) demonstrated that gastrointestinal cancer patients with elevated PNI levels had significantly longer OS (HR: 0.530, 95% CI: 0.456–0.616, *p* < 0.001) and PFS (HR: 0.740, 95% CI: 0.649–0.844, *p* < 0.001), as well as higher objective response rates (OR: 1.622, 95% CI: 1.251–2.103, *p* < 0.004) and disease control rates (OR: 1.846, 95% CI: 1.428–2.388, *p* < 0.001). Similar findings have been reported in oral cancer (25), gynecological cancer (19), prostate cancer (32), and NPC (23, 24). Taken together, these findings validate PNI as a stable, easily measurable screening marker for mortality risk stratification in non-metastatic NPC, with strong prognostic reliability regardless of baseline muscle mass.

The interplay between skeletal muscle mass and nutritional/immune status has emerged as a critical determinant of cancer outcomes. The joint association results provide a compelling argument for the biological “double hit” mechanism in NPC and other solid tumors, where muscle loss combined with nutritional/immune impairment amplifies mortality risk far more than either factor alone (33). Biologically, skeletal muscle serves as the body’s largest labile amino acid reservoir, critical for immune cell proliferation and hepatic acute-phase protein synthesis. In patients with high PNI, their adequate nutritional and immune reserves may act as a “metabolic buffer,” effectively neutralizing the potential hazards of low muscle mass. This explains our “paradoxical” finding where the low eSMI & high PNI group showed no increased risk (HR = 0.96). In contrast, low PNI depletes such reserves, forcing the body to rely on muscle-derived amino acids for homeostasis. Combined with reduced muscle, this final reserve is lost, triggering systemic inflammation and cancer cachexia. Tumor-induced enhanced protein catabolism for tumor proliferation reduces skeletal muscle protein supply, directly causing muscle wasting (29). Additionally, tumor-associated proinflammatory cytokines exacerbate this vicious cycle, simultaneously inducing muscle protein degradation and suppressing albumin synthesis, ultimately amplifying mortality risk (34).

In subgroup analysis, our joint results reveal a critical interaction with treatment intensity. The synergistic risk was prominent in the non-CCRT group (HR = 3.12, *p* = 0.001) but was blunted in those receiving CCRT (*p* = 0.475). This possibly suggests a “treatment masking” effect, where the potent locoregional and systemic control of standard CCRT overriding baseline host biological deficits (29, 35). For patients receiving such aggressive therapy, the therapeutic force may temporarily level survival outcomes regardless of baseline metabolic status. Conversely, in patients receiving less intensive therapy, the host’s endogenous reserves—SMI and PNI—become the primary determinants of resilience (7). This emphasizes that host status is most prognostic when external therapeutic support is less maximized, highlighting the need for proactive nutritional support to improve tolerance for such therapies.

Win ratio analysis was employed as a novel complementary tool to strengthen the clinical reliability of our mortality risk findings. The standard Cox model is limited to single endpoints and fails to fully reflect hierarchical clinical outcomes in event times. By contrast, win ratio analysis prioritizes patient-centered hierarchical outcomes (27, 28, 36)—with mortality as the primary endpoint in our study—and quantifying the likelihood of a superior clinical trajectory between groups. Our win ratio of 1.547 indicates that patients with normal eSMI and high PNI were 54.7% more likely to experience favorable clinical outcomes (no death or delayed progression) than those with dual low eSMI and PNI. This corresponded to a net benefit of 0.159, meaning patients with normal eSMI and high PNI had a 15.9% net advantage in achieving favorable hierarchical clinical outcomes over those with dual low eSMI and PNI. This result confirms the synergistic risk is an important clinical finding rather than a statistical artifact and offers a new statistical approach for future NPC studies with hierarchical endpoints.

Several limitations should be acknowledged. First, eSMI was calculated using anthropometric data rather than using the gold standard CT or DXA imaging. While it may introduce minor measurement error in muscle mass quantification, previous studies have shown a strong agreement between ASM obtained through this equation and the one derived from DEXA (20, 37). Also, anthropometric formula has been successfully applied in recent cancer cohort studies to identify sarcopenia (38). Second, this single-center retrospective design may introduce selection bias, limiting the generalizability of our findings to geographically or ethnically diverse NPC patients. Third, the observational design of the study precludes causal inferences, and residual confounding may persist despite rigorous adjustment for potential confounders. Interventional studies exploring the efficacy of combined strategies targeting muscle preservation and nutritional/immune optimization are warranted to determine whether these approaches can synergistically improve survival outcomes in NPC patients.

## Conclusion

In conclusion, concurrent low muscle mass and immunonutritional depletion identifies a high-risk group of non-metastatic NPC patients at critically elevated mortality risk, with a more than two-fold increase in all-cause mortality. This synergistic hazard highlights the value of routine pre-treatment screening using both simple, readily available markers. Targeted nutritional support and muscle-preserving interventions should be prioritized for these high-risk individuals to improve their long-term survival and clinical outcomes. Future multi-center studies are warranted to validate these findings, explore underlying mechanisms, and evaluate the efficacy of targeted supportive care strategies.

## Data Availability

The raw data supporting the conclusions of this article will be made available by the authors without undue reservation.

## References

[1] Wong KCW Hui EP Lo KW Lam WKJ Johnson D Li L . Nasopharyngeal carcinoma: an evolving paradigm. Nat Rev Clin Oncol. (2021) 18:679–95. doi: 10.1038/s41571-021-00524-x, 34194007

[2] Chen B Zhan Z Xu Y Yu S Huang J Fang Y . Long-term trends in the burden of nasopharyngeal carcinoma in China: a comprehensive analysis from 1990 to 2021 and projections to 2030 based on the global burden of disease study 2021. Radiother Oncol. (2025) 202:110613. doi: 10.1016/j.radonc.2024.110613, 39489428

[3] Liu Q Wang H Chen Z Xiong J Huang Y Zhang S . Global, regional, and national epidemiology of nasopharyngeal carcinoma in middle-aged and elderly patients from 1990 to 2021. Ageing Res Rev. (2024) 104:102613. doi: 10.1016/j.arr.2024.102613, 39626854

[4] Wang H Zhan Y Luo J Wang W Fan S. Unveiling immune resistance mechanisms in nasopharyngeal carcinoma and emerging targets for antitumor immune response: tertiary lymphoid structures. J Transl Med. (2025) 23:38. doi: 10.1186/s12967-024-05880-7, 39789621 PMC11721552

[5] Song J Yang X Wu J Wu Z Niu S Zhuo L . The association analysis between fatigue and body composition loss in patients with nasopharyngeal carcinoma during radiotherapy: an observational longitudinal study. Radiother Oncol. (2024) 197:110340. doi: 10.1016/j.radonc.2024.110340, 38797492

[6] Miao J Wang L Ong EHW Hu C Lin S Chen X . Effects of induction chemotherapy on nutrition status in locally advanced nasopharyngeal carcinoma: a multicentre prospective study. J Cachexia Sarcopenia Muscle. (2023) 14:815–25. doi: 10.1002/jcsm.13196, 36872457 PMC10067484

[7] Huang S Piao Y Cao C Chen J Sheng W Shu Z . A prospective randomized controlled trial on the value of prophylactic oral nutritional supplementation in locally advanced nasopharyngeal carcinoma patients receiving chemo-radiotherapy. Oral Oncol. (2020) 111:105025. doi: 10.1016/j.oraloncology.2020.105025, 33032180

[8] Martin L Senesse P Gioulbasanis I Antoun S Bozzetti F Deans C . Diagnostic criteria for the classification of Cancer-associated weight loss. J Clin Oncol. (2015) 33:90–9. doi: 10.1200/JCO.2014.56.1894, 25422490

[9] Choi MH Yoon SB Lee K Song M Lee IS Lee MA . Preoperative sarcopenia and post-operative accelerated muscle loss negatively impact survival after resection of pancreatic cancer. J Cachexia Sarcopenia Muscle. (2018) 9:326–34. doi: 10.1002/jcsm.12274, 29399990 PMC5879976

[10] Min JH Yu JI Kim SH Kim YK Kim K Park HC . Skeletal muscle index changes on locoregional treatment application after folfirinox and survival in pancreatic cancer. J Cachexia Sarcopenia Muscle. (2025) 16:e16343. doi: 10.1002/jcsm.13643, 39578950 PMC11670158

[11] Wang F Zhen H Yu K Liu P. The prognostic value of sarcopenia in clinical outcomes in cervical cancer: a systematic review and meta-analysis. J Cachexia Sarcopenia Muscle. (2025) 16:e13674. doi: 10.1002/jcsm.13674, 39797562 PMC11724193

[12] Tran SD Forrest NJ Guggilla V Perottino GM Johnson JL Sosman J . Weight and blood-based markers of cachexia predict disability, hospitalization and worse survival in cancer immunotherapy patients. J Cachexia Sarcopenia Muscle. (2025) 16:e13685. doi: 10.1002/jcsm.13685, 39817619 PMC11736629

[13] Chang WP. Relationship between changes in nutritional status during treatment and overall survival of newly diagnosed nasopharyngeal carcinoma patients. Eur J Oncol Nurs. (2024) 73:102721. doi: 10.1016/j.ejon.2024.102721, 39520762

[14] Chen Z Ling J Zhang S Feng Y Xie Y Liu X . Predicting the overall survival and progression-free survival of nasopharyngeal carcinoma patients based on Hemoglobin, albumin, and globulin ratio and classical clinicopathological parameters. Head Neck. (2024) 46:2600–15. doi: 10.1002/hed.27777, 38646952

[15] Herrera-Martinez AD Prior-Sanchez I Fernandez-Soto ML Garcia-Olivares M Novo-Rodriguez C Gonzalez-Pacheco M . Improving the nutritional evaluation in head neck cancer patients using bioelectrical impedance analysis: not only the phase angle matters. J Cachexia Sarcopenia Muscle. (2024) 15:2426–36. doi: 10.1002/jcsm.13577, 39447215 PMC11634526

[16] He WZ Jiang C Liu LL Yin CX Rong YM Hu WM . Association of body composition with survival and inflammatory responses in patients with non-metastatic nasopharyngeal cancer. Oral Oncol. (2020) 108:104771. doi: 10.1016/j.oraloncology.2020.104771, 32485608

[17] Muscaritoli M Anker SD Argiles J Aversa Z Bauer JM Biolo G . Consensus definition of sarcopenia, cachexia and pre-cachexia: joint document elaborated by special interest groups (sig) "cachexia-anorexia in chronic wasting diseases" and "nutrition in geriatrics". Clin Nutr. (2010) 29:154–9. doi: 10.1016/j.clnu.2009.12.004, 20060626

[18] Fearon K Strasser F Anker SD Bosaeus I Bruera E Fainsinger RL . Definition and classification of cancer cachexia: an international consensus. Lancet Oncol. (2011) 12:489–95. doi: 10.1016/S1470-2045(10)70218-7, 21296615

[19] Wang X Wang Y. The prognostic nutritional index is prognostic factor of gynecological cancer: a systematic review and meta-analysis. Int J Surg. (2019) 67:79–86. doi: 10.1016/j.ijsu.2019.05.018, 31185310

[20] Wen X Wang M Jiang CM Zhang YM. Anthropometric equation for estimation of appendicular skeletal muscle mass in Chinese adults. Asia Pac J Clin Nutr. (2011) 20:551–6. 22094840

[21] Zhang L Ma W Qiu Z Kuang T Wang K Hu B . Prognostic nutritional index as a prognostic biomarker for gastrointestinal cancer patients treated with immune checkpoint inhibitors. Front Immunol. (2023) 14:1219929. doi: 10.3389/fimmu.2023.1219929, 37545502 PMC10401046

[22] Chen Z Zeng L Cai W Song X Xu Q Xu J . Predictive value of three nutritional indexes for disease activity in patients with inflammatory bowel disease. Ann Med. (2025) 57:2443256. doi: 10.1080/07853890.2024.2443256, 39705015 PMC11703468

[23] Tu X Ren J Zhao Y. Prognostic value of prognostic nutritional index in nasopharyngeal carcinoma: a meta-analysis containing 4511 patients. Oral Oncol. (2020) 110:104991. doi: 10.1016/j.oraloncology.2020.104991, 32919361

[24] Kucukarda A Erdogan B Gokyer A Sayin S Gokmen I Ozcan E . Prognostic nutritional index and its dynamics after curative treatment are independent prognostic factors on survival in non-metastatic nasopharyngeal carcinoma. Support Care Cancer. (2022) 30:2131–9. doi: 10.1007/s00520-021-06627-6, 34677649

[25] Kubota K Ito R Narita N Tanaka Y Furudate K Akiyama N . Utility of prognostic nutritional index and systemic immune-inflammation index in oral cancer treatment. BMC Cancer. (2022) 22:368. doi: 10.1186/s12885-022-09439-x, 35392843 PMC8991673

[26] Coin A Sarti S Ruggiero E Giannini S Pedrazzoni M Minisola S . Prevalence of sarcopenia based on different diagnostic criteria using dexa and appendicular skeletal muscle mass reference values in an Italian population aged 20 to 80. J Am Med Dir Assoc. (2013) 14:507–12. doi: 10.1016/j.jamda.2013.02.010, 23582341

[27] Cunningham JM Fuest S Burke H. Win ratio method for hierarchical composite outcomes in randomized clinical trials. JAMA. (2026) 335:715–6. doi: 10.1001/jama.2025.25686, 41604184

[28] Wang D Pocock S. A win ratio approach to comparing continuous non-normal outcomes in clinical trials. Pharm Stat. (2016) 15:238–45. doi: 10.1002/pst.1743, 26970432

[29] Liu S Zou Y Zhong M Li T Cao Y Wang R . Prognostic significance of MRI-defined sarcopenia in patients with nasopharyngeal carcinoma: a propensity score matched analysis of real-world data. Radiother Oncol. (2023) 188:109904. doi: 10.1016/j.radonc.2023.109904, 37678624

[30] Ucgul E Guven DC Ucgul AN Ozbay Y Onur MR Akin S. Factors influencing immunotherapy outcomes in cancer: sarcopenia and systemic inflammation. Cancer Control. (2024) 31:10732748241302248. doi: 10.1177/10732748241302248, 39547932 PMC11569492

[31] Wiegert EVM de Oliveira LC Calixto-Lima L Borges NA Rodrigues J da Mota ESLMS . Association between low muscle mass and survival in incurable cancer patients: a systematic review. Nutrition. (2020) 72:110695. doi: 10.1016/j.nut.2019.110695, 32007806

[32] Zheng Y Wang K Ou Y Hu X Wang Z Wang D . Prognostic value of a baseline prognostic nutritional index for patients with prostate cancer: a systematic review and meta-analysis. Prostate Cancer Prostatic Dis. (2024) 27:604–13. doi: 10.1038/s41391-023-00689-9, 37391595

[33] Wang X Yang M Ge Y Tang M Rao B Chen Y . Association of systemic inflammation and malnutrition with survival in nasopharyngeal carcinoma undergoing chemoradiotherapy: results from a multicenter cohort study. Front Oncol. (2021) 11:766398. doi: 10.3389/fonc.2021.766398, 34765561 PMC8576523

[34] Argiles JM Busquets S Felipe A Lopez-Soriano FJ. Molecular mechanisms involved in muscle wasting in cancer and ageing: cachexia versus sarcopenia. Int J Biochem Cell Biol. (2005) 37:1084–104. doi: 10.1016/j.biocel.2004.10.003, 15743680

[35] Thureau S Lebret L Lequesne J Cabourg M Dandoy S Gouley C . Prospective evaluation of sarcopenia in head and neck cancer patients treated with radiotherapy or radiochemotherapy. Cancers (Basel). (2021) 13:753. doi: 10.3390/cancers13040753, 33670339 PMC7917983

[36] Pocock SJ Ariti CA Collier TJ Wang D. The win ratio: a new approach to the analysis of composite endpoints in clinical trials based on clinical priorities. Eur Heart J. (2012) 33:176–82. doi: 10.1093/eurheartj/ehr352, 21900289

[37] Luo YX Zhou XH Heng T Yang LL Zhu YH Hu P . Bidirectional transitions of sarcopenia states in older adults: the longitudinal evidence from CHARLS. J Cachexia Sarcopenia Muscle. (2024) 15:1915–29. doi: 10.1002/jcsm.13541, 39001569 PMC11446714

[38] Santos TG da Silva LL Bernabe RAM Albergaria BH Machado JM Marques-Rocha JL . Anthropometric equation has sufficient diagnostic capacity to identify sarcopenia in women with breast cancer. BMC Cancer. (2024) 24:1310. doi: 10.1186/s12885-024-12921-3, 39448968 PMC11515378

