# Supplementary material for: Joint association of estimated skeletal muscle mass index and prognostic nutritional index with all-cause mortality in individuals with non-metastatic nasopharyngeal cancer
Source: Front Nutr. 2026 May 14;13:1768802. doi: 10.3389/fnut.2026.1768802 (PMC13215891; doi:10.3389/fnut.2026.1768802)
Supplement: Supplementary file 1 [file Data_sheet_1.docx]

**Joint association of estimated skeletal muscle mass index and prognostic nutritional index with all-cause mortality in individuals with non-metastatic nasopharyngeal cancer**

**Supplementary files**

**Supplementary** **Table S1** Characteristics of the population stratified by eSMI based on sex-specific P20

**Supplementary Table S2** Characteristics of the population by PNI levels

**Supplementary Table S3** Inverse Probability of Treatment Weighting-adjusted win ratio analysis

**Supplementary Table S4** The subgroup analysis of association of eSMI, PNI and combined stratification group with all-cause mortality

**Supplementary Table S1.** Characteristics of the population stratified by eSMI based on sex-specific P20

| **Characteristic of baseline** | **Overall  N = 942** | **Normal eSMI  N = 752** | **Low eSMI N = 190** | **P-value ^1^** |
| --- | --- | --- | --- | --- |
| Male | 666 (71%) | 532 (71%) | 134 (71%) | 0.953 |
| Age, years | 49 ± 10 | 48 ± 10 | 52 ± 10 | <0.001 |
| Age group |  |  |  | <0.001 |
| ˂ 45 years | 306 (32%) | 264 (35%) | 42 (22%) |  |
| ≥ 45 years | 636 (68%) | 488 (65%) | 148 (78%) |  |
| Current smoking | 229 (24%) | 183 (24%) | 46 (24%) | 0.971 |
| Current drinking | 141 (15%) | 108 (14%) | 33 (17%) | 0.299 |
| Family history of cancers | 30 (3.2%) | 26 (3.5%) | 4 (2.1%) | 0.343 |
| Comorbidity of chronic diseases^2^ | 348 (37%) | 285 (38%) | 63 (33%) | 0.226 |
| T stage |  |  |  | 0.901 |
| 1~2 | 492 (52%) | 392 (52%) | 100 (53%) |  |
| 3~4 | 450 (48%) | 360 (48%) | 90 (47%) |  |
| N stage |  |  |  | 0.269 |
| 0~1 | 222 (24%) | 183 (24%) | 39 (21%) |  |
| 2~3 | 720 (76%) | 569 (76%) | 151 (79%) |  |
| Clinical stage |  |  |  | 0.081 |
| I-II | 131 (14%) | 106 (14%) | 25 (13%) |  |
| III | 583 (62%) | 453 (60%) | 130 (68%) |  |
| IV | 228 (24%) | 193 (26%) | 35 (18%) |  |
| OS, months | 54 (37, 75) | 54 (37, 77) | 56 (36, 72) | 0.709 |
| CCRT | 289 (31%) | 237 (32%) | 52 (27%) | 0.268 |
| PS score |  |  |  | <0.001 |
| 0 | 548 (58%) | 461 (61%) | 87 (46%) |  |
| 1~2 | 394 (42%) | 291 (39%) | 103 (54%) |  |
| BMI, kg/m^2^ | 23.4 ± 3.2 | 24.4 ± 2.6 | 19.2 ± 1.4 | <0.001 |
| BMI group |  |  |  | <0.001 |
| ˂ 24 kg/m^2^ | 547 (58%) | 357 (47%) | 190 (100%) |  |
| ≥ 24 kg/m^2^ | 395 (42%) | 395 (53%) | 0 (0%) |  |
| Hemoglobin, g/L | 142 ± 16 | 143 ± 15 | 136 ± 15 | <0.001 |
| SMI, kg/m^2^ | 7.38 ± 0.94 | 7.60 ± 0.84 | 6.51 ± 0.76 | <0.001 |
| PNI | 50.3 ± 4.7 | 50.5 ± 4.5 | 49.3 ± 5.0 | <0.001 |
| PNI group |  |  |  | <0.001 |
| ≥ 45 | 830 (88%) | 677 (90%) | 153 (81%) |  |
| ˂ 45 | 112 (12%) | 75 (10.0%) | 37 (19%) |  |
| Albumin, g/L | 41.8 ± 3.5 | 42.0 ± 3.4 | 40.9 ± 3.9 | <0.001 |
| Albumin group |  |  |  | <0.001 |
| ˂ 40 g/L | 271 (29%) | 197 (26%) | 74 (39%) |  |
| ≥ 40 g/L | 671 (71%) | 555 (74%) | 116 (61%) |  |
| Leukocyte, 10^9^/L | 6.60 ± 1.89 | 6.63 ± 1.88 | 6.49 ± 1.96 | 0.186 |
| Neutrophil, 10^9^/L | 4.37 ± 1.66 | 4.39 ± 1.61 | 4.30 ± 1.82 | 0.129 |
| Lymphocyte, 10^9^/L | 1.70 ± 0.58 | 1.71 ± 0.57 | 1.67 ± 0.60 | 0.188 |

The continuous data were presented as the mean ±SD or median (IQR) as appropriate. The categorical data were presented as absolute numbers and percentages (%) of the total.

^1^ Pearson's Chi-squared test, Kruskal-Wallis rank sum test or Fisher's exact test were used as appropriate.

^2^ Comorbidity of chronic diseases was determined by the histories of hypertension, diabetes, or HBV infection.

Abbreviations: BMI, body mass index; n, number; CCRT, concurrent chemoradiotherapy; eSMI, estimated skeletal muscle index; HBV, hepatitis B virus; OS, overall survival; PNI, prognostic nutritional index; PS, performance status; SD, standard deviation.

**Supplementary Table S2.** Characteristics of the population by PNI levels

| **Characteristic of baseline** | **Overall  N = 942** | **PNI ≥ 45  N = 830** | **PNI < 45  N = 112** | **P-value^1^** |
| --- | --- | --- | --- | --- |
| Male | 666 (71%) | 586 (71%) | 80 (71%) | 0.857 |
| Age | 49 ± 10 | 49 ± 10 | 49 ± 10 | 0.566 |
| Age group |  |  |  | 0.609 |
| ˂ 45 years | 306 (32%) | 272 (33%) | 34 (30%) |  |
| ≥ 45 years | 636 (68%) | 558 (67%) | 78 (70%) |  |
| Current smoking | 229 (24%) | 199 (24%) | 30 (27%) | 0.515 |
| Current drinking | 141 (15%) | 123 (15%) | 18 (16%) | 0.727 |
| Family history of cancers | 30 (3.2%) | 27 (3.3%) | 3 (2.7%) | >0.999 |
| Comorbidity of chronic diseases^2^ | 348 (37%) | 311 (37%) | 37 (33%) | 0.361 |
| T stage |  |  |  | 0.087 |
| 1~2 | 492 (52%) | 442 (53%) | 50 (45%) |  |
| 3~4 | 450 (48%) | 388 (47%) | 62 (55%) |  |
| N stage |  |  |  | 0.925 |
| 0~1 | 222 (24%) | 196 (24%) | 26 (23%) |  |
| 2~3 | 720 (76%) | 634 (76%) | 86 (77%) |  |
| Clinical stage |  |  |  | 0.525 |
| I-II | 131 (14%) | 119 (14%) | 12 (11%) |  |
| III | 583 (62%) | 513 (62%) | 70 (63%) |  |
| IV | 228 (24%) | 198 (24%) | 30 (27%) |  |
| OS, months | 54 (37, 75) | 55 (38, 76) | 50 (33, 73) | 0.144 |
| CCRT | 289 (31%) | 259 (31%) | 30 (27%) | 0.341 |
| PS score |  |  |  | <0.001 |
| 0 | 548 (58%) | 499 (60%) | 49 (44%) |  |
| 1~2 | 394 (42%) | 331 (40%) | 63 (56%) |  |
| BMI, kg/m^2^ | 23.4 ± 3.2 | 23.6 ± 3.2 | 22.2 ± 3.2 | <0.001 |
| BMI group |  |  |  | 0.001 |
| ˂ 24 kg/m^2^ | 547 (58%) | 466 (56%) | 81 (72%) |  |
| ≥ 24 kg/m^2^ | 395 (42%) | 364 (44%) | 31 (28%) |  |
| Hemoglobin, g/L | 142 ± 16 | 143 ± 15 | 128 ± 13 | <0.001 |
| SMI, kg/m^2^ | 7.38 ± 0.94 | 7.41 ± 0.94 | 7.15 ± 0.90 | 0.006 |
| SMI group |  |  |  | <0.001 |
| Normal | 752 (80%) | 677 (82%) | 75 (67%) |  |
| Low | 190 (20%) | 153 (18%) | 37 (33%) |  |
| PNI | 50.3 ± 4.7 | 51.3 ± 3.8 | 42.5 ± 2.5 | <0.001 |
| Albumin, g/L | 41.8 ± 3.5 | 42.5 ± 2.9 | 36.3 ± 2.3 | <0.001 |
| Albumin group |  |  |  | <0.001 |
| ˂ 40 g/L | 271 (29%) | 162 (20%) | 109 (97%) |  |
| ≥ 40 g/L | 671 (71%) | 668 (80%) | 3 (2.7%) |  |
| Leukocyte, 10^9^/L | 6.60 ± 1.89 | 6.68 ± 1.85 | 6.06 ± 2.11 | <0.001 |
| Neutrophil, 10^9^/L | 4.37 ± 1.66 | 4.38 ± 1.60 | 4.28 ± 2.01 | 0.090 |
| Lymphocyte, 10^9^/L | 1.70 ± 0.58 | 1.76 ± 0.57 | 1.25 ± 0.36 | <0.001 |

The continuous data were presented as the mean ±SD or median (IQR) as appropriate. The categorical data were presented as absolute numbers and percentages (%) of the total.

^1^ Pearson's Chi-squared test, Kruskal-Wallis rank sum test or Fisher's exact test were used as appropriate.

^2^ Comorbidity of chronic diseases was determined by the histories of hypertension, diabetes, or HBV infection.

Abbreviations: BMI, body mass index; n, number; CCRT, concurrent chemoradiotherapy; HBV, hepatitis B virus; OS, overall survival; PNI, prognostic nutritional index; PS, performance status; SD, standard deviation; SMI, skeletal muscle index.

**Supplementary Table S3.** Inverse Probability of Treatment Weighting-adjusted win ratio analysis

| **Outcome Metric** | **Estimate** | | **95% Confidence Interval** | **P Value** |
| --- | --- | --- | --- | --- |
| Win Ratio (Death > Progression) | 1.5470 | | (1.536,1.557) | **<0.0001** |
| Win Odds (Including Ties) | 1.3790 | | (1.371,1.387) | **<0.0001** |
| Net Benefit | 0.1593 | | - | **<0.0001** |
| **Weighted Metric** | | **Value** | | |
| Total Weighted Pairs | | 463,667.6 | | |
| Weighted Wins (Low Risk Group) | | 208,942.8 | | |
| Weighted Wins (High Risk Group) | | 135,077.8 | | |
| Weighted Ties | | 119,638.4 | | |

Inverse Probability of Treatment Weighting-adjusted comparison of clinical outcomes between llow-risk (normal eSMI & high PNI) and high-risk (low eSMI & low PNI) groups (death as primary outcome, progression as secondary outcome).

**Table S4. The subgroup analysis of association of eSMI, PNI and combined stratification group with all-cause mortality**

| **Subgroup** | **Crude model** | | **Model 1** | | **Model 2** | |
| --- | --- | --- | --- | --- | --- | --- |
|  | **95%CI** | **P** | **95%CI** | **P** | **95%CI** | **P** |
| **Age ≥ 45 years** |  |  |  |  |  |  |
| **eSMI, kg/m^2^** | 0.78(0.66,0.94) | 0.008 | 0.9(0.68,1.18) | 0.439 | 0.3(0.04, 2.03) | 0.216 |
| **eSMI group** |  |  |  |  |  |  |
| Normal | ref |  | ref |  | ref |  |
| Low | 1.24(0.85,1.80) | 0.270 | 1.22(0.83,1.78) | 0.306 | 1.2(0.70,2.07) | 0.511 |
| **PNI** | 0.93(0.90,0.96) | <0.001 | 0.93(0.90,0.96) | <0.001 | 0.93(0.89, 0.97) | <0.001 |
| **PNI** |  |  |  |  |  |  |
| ≥ 45 | ref |  | ref |  | ref |  |
| ˂ 45 | 1.99(1.32,3.02) | 0.001 | 1.98(1.31,3.01) | 0.001 | 1.94(1.26, 2.99) | 0.003 |
| **Joint group** |  |  |  |  |  |  |
| Normal eSMI & high PNI | ref |  | ref |  | ref |  |
| Normal eSMI & low PNI | 1.91(1.13,3.22) | 0.015 | 1.93(1.15,3.26) | 0.014 | 2.07(1.20,3.57) | 0.009 |
| Low eSMI & high PNI | 1.14(0.73,1.77) | 0.556 | 1.13(0.73,1.76) | 0.585 | 1.31(0.78,2.20) | 0.308 |
| Low eSMI & low PNI | 2.33(1.24,4.38) | 0.009 | 2.23(1.18,4.22) | 0.013 | 2.69(1.33,5.41) | 0.006 |
| P for trend |  | 0.034 |  | 0.041 |  | 0.013 |
| **Age < 45 years** |  |  |  |  |  |  |
| **eSMI, kg/m^2^** | 1.08(0.84,1.38) | 0.554 | 1.19(0.83,1.71) | 0.337 | 12.14(0.24,619.57) | 0.214 |
| **eSMI group** |  |  |  |  |  |  |
| Normal | ref |  | ref |  | ref |  |
| Low | 0.53(0.21,1.32) | 0.172 | 0.54(0.22,1.34) | 0.183 | 0.55(0.19,1.61) | 0.274 |
| **PNI** | 1.03(0.98,1.08) | 0.31 | 1.02(0.98,1.07) | 0.342 | 1.03(0.97, 1.08) | 0.352 |
| **PNI** |  |  |  |  |  |  |
| ≥ 45 | ref |  | ref |  | ref |  |
| ˂ 45 | 1.18(0.56,2.47) | 0.66 | 1.18(0.56,2.48) | 0.655 | 0.93(0.40, 2.16) | 0.866 |
| **Joint group** |  |  |  |  |  |  |
| Normal eSMI & high PNI | ref |  | ref |  | ref |  |
| Normal eSMI & low PNI | 0.85(0.34,2.11) | 0.72 | 0.84(0.34,2.10) | 0.714 | 0.78(0.29,2.08) | 0.614 |
| Low eSMI & high PNI | 0.25(0.06,1.02) | 0.054 | 0.25(0.06,1.03) | 0.054 | 0.27(0.06,1.15) | 0.076 |
| Low eSMI & low PNI | 1.93(0.60,6.17) | 0.27 | 2.07(0.64,6.73) | 0.228 | 2.32(0.66,8.16) | 0.19 |
| P for trend |  | 0.294 |  | 0.312 |  | 0.45 |
| **BMI ≥ 24 kg/m^2 #^** |  |  |  |  |  |  |
| **eSMI, kg/m^2^** | 0.69(0.54,0.89) | 0.004 | 0.77(0.44,1.36) | 0.366 | 0.8(0.45,1.42) | 0.446 |
| **PNI** | 0.94(0.90,0.98) | 0.006 | 0.94(0.90,0.98) | 0.005 | 0.93(0.89,0.97) | 0.002 |
| **PNI** |  |  |  |  |  |  |
| ≥ 45 | ref |  | ref |  | ref |  |
| ˂ 45 | 2.64(1.51,4.60) | <0.001 | 2.63(1.51,4.59) | <0.001 | 3.3(1.81,6.02) | <0.001 |
| **BMI < 24 kg/m^2^** |  |  |  |  |  |  |
| **eSMI, kg/m^2^** | 0.94(0.75,1.17) | 0.565 | 0.92(0.56,1.51) | 0.753 | 0.95(0.57,1.59) | 0.856 |
| **eSMI group** |  |  |  |  |  |  |
| Normal | ref |  | ref |  | ref |  |
| Low | 1.11(0.76,1.63) | 0.586 | 1.13(0.76,1.67) | 0.545 | 1.11(0.74,1.66) | 0.625 |
| **PNI** | 0.98(0.94,1.02) | 0.246 | 0.98(0.94,1.02) | 0.244 | 0.98(0.94,1.02) | 0.297 |
| **PNI** |  |  |  |  |  |  |
| ≥ 45 | ref |  | ref |  | ref |  |
| ˂ 45 | 1.38(0.86,2.22) | 0.179 | 1.39(0.86,2.23) | 0.175 | 1.36(0.84,2.20) | 0.217 |
| **Joint group** |  |  |  |  |  |  |
| Normal eSMI & high PNI | ref |  | ref |  | ref |  |
| Normal eSMI & low PNI | 0.76(0.35,1.66) | 0.497 | 0.77(0.35,1.67) | 0.501 | 0.79(0.35,1.74) | 0.552 |
| Low eSMI & high PNI | 0.87(0.56,1.35) | 0.53 | 0.88(0.56,1.38) | 0.575 | 0.93(0.59,1.48) | 0.765 |
| Low eSMI & low PNI | 2.1(1.18,3.76) | 0.012 | 2.14(1.19,3.85) | 0.011 | 2.31(1.27,4.18) | 0.006 |
| P for trend |  | 0.341 |  | 0.308 |  | 0.184 |
| **Comorbidity = yes** |  |  |  |  |  |  |
| **eSMI, kg/m^2^** | 0.83(0.66,1.05) | 0.121 | 0.85(0.60,1.21) | 0.36 | 1.16(0.06,21.19) | 0.921 |
| **eSMI group** |  |  |  |  |  |  |
| Normal | ref |  | ref |  | ref |  |
| Low | 1.17(0.68,2.03) | 0.567 | 1.27(0.73,2.21) | 0.403 | 0.95(0.44,2.06) | 0.89 |
| **PNI** | 0.95(0.90,0.99) | 0.024 | 0.94(0.90,0.99) | 0.017 | 0.94(0.90, 0.99) | 0.015 |
| **PNI** |  |  |  |  |  |  |
| ≥ 45 | ref |  | ref |  | ref |  |
| ˂ 45 | 2.05(1.15,3.65) | 0.015 | 2.07(1.16,3.72) | 0.014 | 2.62(1.40, 4.89) | 0.003 |
| **Joint group** |  |  |  |  |  |  |
| Normal eSMI & high PNI | ref |  | ref |  | ref |  |
| Normal eSMI & low PNI | 1.89(0.96,3.72) | 0.066 | 1.87(0.94,3.72) | 0.074 | 2.26(1.11, 4.60) | 0.025 |
| Low eSMI & high PNI | 1.07(0.57,2.01) | 0.825 | 1.15(0.61,2.18) | 0.656 | 1.12(0.55, 2.26) | 0.762 |
| Low eSMI & low PNI | 2.74(0.99,7.57) | 0.052 | 3.2(1.15,8.93) | 0.026 | 5.29(1.67,16.81) | 0.005 |
| P for trend |  | 0.161 |  | 0.093 |  | 0.072 |
| **Comorbidity = no** |  |  |  |  |  |  |
| **eSMI, kg/m^2^** | 0.91(0.75,1.09) | 0.305 | 1.11(0.83,1.47) | 0.481 | 0.24(0.03, 1.83) | 0.167 |
| **eSMI group** |  |  |  |  |  |  |
| Normal | ref |  | ref |  | ref |  |
| Low | 0.98(0.64,1.51) | 0.925 | 0.93(0.60,1.45) | 0.76 | 1.11(0.62,1.98) | 0.735 |
| **PNI** | 0.97(0.93,1.01) | 0.137 | 0.97(0.93,1.01) | 0.118 | 0.96(0.92, 1.00) | 0.062 |
| **PNI** |  |  |  |  |  |  |
| ≥ 45 | ref |  | ref |  | ref |  |
| ˂ 45 | 1.54(0.97,2.44) | 0.066 | 1.59(1.00,2.53) | 0.048 | 1.65(1.02, 2.69) | 0.042 |
| **Joint group** |  |  |  |  |  |  |
| Normal eSMI & high PNI | ref |  | ref |  | ref |  |
| Normal eSMI & low PNI | 1.22(0.67,2.23) | 0.524 | 1.29(0.70,2.37) | 0.409 | 1.35(0.72,2.54) | 0.357 |
| Low eSMI & high PNI | 0.77(0.45,1.31) | 0.331 | 0.73(0.43,1.26) | 0.26 | 0.85(0.47,1.54) | 0.593 |
| Low eSMI & low PNI | 1.96(1.01,3.78) | 0.045 | 1.89(0.97,3.66) | 0.06 | 2.18(1.06,4.45) | 0.033 |
| P for trend |  | 0.566 |  | 0.671 |  | 0.291 |
| **CCRT = yes** |  |  |  |  |  |  |
| **eSMI, kg/m^2^** | 0.91(0.66,1.25) | 0.553 | 0.83(0.52,1.31) | 0.415 | 0.13(0.00, 11.46) | 0.367 |
| **eSMI group** |  |  |  |  |  |  |
| Normal | ref |  | ref |  | ref |  |
| Low | 0.89(0.42,1.92) | 0.77 | 0.97(0.45,2.11) | 0.945 | 0.56(0.21,1.51) | 0.254 |
| **PNI** | 0.97(0.91,1.03) | 0.369 | 0.97(0.91,1.03) | 0.346 | 0.98(0.92, 1.05) | 0.653 |
| **PNI** |  |  |  |  |  |  |
| ≥ 45 | ref |  | ref |  | ref |  |
| ˂ 45 | 1.04(0.41,2.64) | 0.931 | 1.04(0.41,2.63) | 0.936 | 0.77(0.28, 2.07) | 0.601 |
| **Joint group** |  |  |  |  |  |  |
| Normal eSMI & high PNI | ref |  | ref |  | ref |  |
| Normal eSMI & low PNI | 0.59(0.14,2.43) | 0.461 | 0.57(0.14,2.36) | 0.438 | 0.52(0.12,2.24) | 0.383 |
| Low eSMI & high PNI | 0.66(0.26,1.68) | 0.38 | 0.72(0.28,1.84) | 0.488 | 0.56(0.20,1.51) | 0.25 |
| Low eSMI & low PNI | 1.78(0.54,5.82) | 0.34 | 1.98(0.60,6.55) | 0.266 | 1.59(0.45,5.69) | 0.475 |
| P for trend |  | 0.817 |  | 0.974 |  | 0.57 |
| **CCRT = no** |  |  |  |  |  |  |
| **eSMI, kg/m^2^** | 0.9(0.76,1.07) | 0.231 | 1.06(0.83,1.37) | 0.636 | 0.67(0.11, 3.99) | 0.66 |
| **eSMI group** |  |  |  |  |  |  |
| Normal | ref |  | ref |  | ref |  |
| Low | 1.07(0.73,1.56) | 0.729 | 1.08(0.73,1.58) | 0.704 | 1.3(0.76,2.21) | 0.332 |
| **PNI** | 0.96(0.93,0.99) | 0.016 | 0.96(0.92,0.99) | 0.011 | 0.95(0.92, 0.98) | 0.005 |
| **PNI** |  |  |  |  |  |  |
| ≥ 45 | ref |  | ref |  | ref |  |
| ˂ 45 | 1.9(1.29,2.82) | 0.001 | 1.94(1.31,2.88) | <0.001 | 2.07(1.37, 3.11) | <0.001 |
| **Joint group** |  |  |  |  |  |  |
| Normal eSMI & high PNI | ref |  | ref |  | ref |  |
| Normal eSMI & low PNI | 1.71(1.06,2.76) | 0.029 | 1.73(1.07,2.79) | 0.026 | 1.96(1.19,3.24) | 0.008 |
| Low eSMI & high PNI | 0.92(0.59,1.45) | 0.733 | 0.93(0.59,1.46) | 0.738 | 1.17(0.70,1.97) | 0.552 |
| Low eSMI & low PNI | 2.29(1.23,4.26) | 0.009 | 2.4(1.29,4.49) | 0.006 | 3.12(1.58,6.17) | 0.001 |
| P for trend |  | 0.141 |  | 0.121 |  | 0.011 |
| **PS score = 0** |  |  |  |  |  |  |
| **eSMI, kg/m^2^** |  |  |  |  |  |  |
| **eSMI group** | ref |  | ref |  | ref |  |
| Normal | 1.19(0.73,1.95) | 0.487 | 1.13(0.68,1.88) | 0.639 | 1.86(0.93, 3.70) | 0.078 |
| Low | 1.65(1.02,2.65) | 0.040 | 1.2(0.57,2.51) | 0.635 | 3.39(1.11,10.39) | 0.033 |
| **PNI** |  | 0.038 |  | 0.6 |  | 0.032 |
| **PNI** |  |  |  |  |  |  |
| ≥ 45 | ref |  | ref |  | ref |  |
| ˂ 45 | 1.15(0.75,1.76) | 0.512 | 1.18(0.77,1.80) | 0.456 | 1.22(0.79,1.88) | 0.365 |
| **Joint group** |  |  |  |  |  |  |
| Normal eSMI & high PNI | ref |  | ref |  | ref |  |
| Normal eSMI & low PNI | 0.67(0.41,1.10) | 0.116 | 0.68(0.41,1.12) | 0.128 | 0.69(0.41,1.15) | 0.157 |
| Low eSMI & high PNI | 0.36(0.12,1.02) | 0.054 | 0.26(0.08,0.82) | 0.022 | 0.37(0.10,1.27) | 0.113 |
| Low eSMI & low PNI | 1.54(0.93,2.55) | 0.092 | 1.14(0.57,2.26) | 0.718 | 1.68(0.73,3.82) | 0.220 |
| P for trend |  | 0.034 |  | 0.51 |  | 0.141 |
| **PS 1~2** |  |  |  |  |  |  |
| **eSMI, kg/m^2^** | 0.91(0.74,1.13) | 0.397 | 0.95(0.70,1.29) | 0.737 | 0.52(0.04, 6.46) | 0.611 |
| **eSMI group** |  |  |  |  |  |  |
| Normal | ref |  | ref |  | ref |  |
| Low | 0.93(0.59,1.46) | 0.747 | 0.95(0.60,1.49) | 0.811 | 0.78(0.40,1.49) | 0.447 |
| **PNI** | 0.96(0.92,1.00) | 0.029 | 0.96(0.92,1.00) | 0.033 | 0.96(0.92, 1.00) | 0.033 |
| **PNI** |  |  |  |  |  |  |
| ≥ 45 | ref |  | ref |  | ref |  |
| ˂ 45 | 1.59(0.98,2.57) | 0.06 | 1.56(0.96,2.52) | 0.073 | 1.52(0.93, 2.51) | 0.073 |
| **Joint group** |  |  |  |  |  |  |
| Normal eSMI & high PNI | ref |  | ref |  | ref |  |
| Normal eSMI & low PNI | 1.5(0.84,2.69) | 0.169 | 1.46(0.82,2.62) | 0.201 | 1.44(0.78,2.66) | 0.201 |
| Low eSMI & high PNI | 0.86(0.51,1.46) | 0.583 | 0.88(0.52,1.49) | 0.626 | 0.97(0.52,1.78) | 0.626 |
| Low eSMI & low PNI | 1.59(0.73,3.48) | 0.245 | 1.61(0.73,3.51) | 0.236 | 1.73(0.74,4.03) | 0.236 |
| P for trend |  | 0.699 |  | 0.656 |  | 0.656 |

^#^Analysis of low eSMI and the joint effect of low eSMI & low PNI was not performed in the BMI ≥ 24 kg/m² subgroup, due to the absence of patients with low eSMI in this BMI stratum (n=0).

Model 1 was adjusted for age and sex.

Model 2 was further adjusted for smoking status, drinking status, comorbidities of chronic diseases, clinical staging, pathological T and N stage, CCRT, PS score, neutrophil, eSMI, PNI, and BMI.

Abbreviations: BMI, body mass index; CCRT, concurrent chemoradiotherapy; eSMI, estimated skeletal muscle index; PNI, prognostic nutritional index; PS, performance status.
